# Anatomy ontologies and potential users: bridging the gap

**DOI:** 10.1186/2041-1480-2-S4-S3

**Published:** 2011-08-09

**Authors:** Ravensara S Travillian, Tomasz Adamusiak, Tony Burdett, Michael Gruenberger, John Hancock, Ann-Marie Mallon, James Malone, Paul Schofield, Helen Parkinson

**Affiliations:** 1 EMBL-EBI, Wellcome Trust Genome Campus, Hinxton, Cambridgeshire CB10 1SD, UK; 2 Department of Physiology, Development, and Neuroscience, University of Cambridge, Downing Street, Cambridge CB2 3DY, UK; 3 MRC Mammalian Genetics Unit, Harwell, Oxfordshire OX11 0RD, UK

## Abstract

**Motivation:**

To evaluate how well current anatomical ontologies fit the way real-world users apply anatomy terms in their data annotations.

**Methods:**

Annotations from three diverse multi-species public-domain datasets provided a set of use cases for matching anatomical terms in two major anatomical ontologies (the Foundational Model of Anatomy and Uberon), using two lexical-matching applications (Zooma and Ontology Mapper).

**Results:**

Approximately 1500 terms were identified; Uberon/Zooma mappings provided 286 matches, compared to the control and Ontology Mapper returned 319 matches. For the Foundational Model of Anatomy, Zooma returned 312 matches, and Ontology Mapper returned 397.

**Conclusions:**

Our results indicate that for our datasets the anatomical entities or concepts are embedded in user-generated complex terms, and while lexical mapping works, anatomy ontologies do not provide the majority of terms users supply when annotating data. Provision of searchable cross-products for compositional terms is a key requirement for using ontologies.

## Background

The need for anatomy ontologies to support researchers and clinicians in managing the explosion of experimental data is well-documented [1]. Four of the 8 Open Biological and Biomedical Ontologies (OBO) Foundry ontologies and 39 of the site’s other listed ontologies, cover aspects of the representation of anatomical knowledge. Whilst the OBO Foundry has a stated goal of “creating a suite of orthogonal interoperable reference ontologies in the biomedical domain” (http://www.obofoundry.org), most of these ontologies have been developed to address a species-specific need articulated by a community working with a particular model organism—for example, ZFIN by the zebrafish researcher community [2], FlyBase by the *Drosophila* researcher community [3], and the various databases dedicated to different ways of representing the needs of mouse researchers [4]. As a result, despite the number of ontologies and the amount of knowledge represented within them, efforts to integrate data across species are in their infancy. We undertook this study—matching terms from real-world data annotations to two major anatomical ontologies—to illuminate some of the existing obstacles to such integration, and to propose ways of making the ontologies more usable to real-world users.

The motivation behind this study was to determine how well current ontologies fit the way users apply anatomy terms in their knowledge domains. We wished to explore whether there was a gap between available ontologies and the use cases for functional genomics data annotation. We also wished to demonstrate our process to show where any gaps and omissions lie.

This paper describes the results of a test of lexical matching anatomical terms entered by current users of an active database of experimental annotation to two major state-of-the-art anatomy ontologies. The implications of our results for the current state of usability for those ontologies in the users’ experience are described. We go on to propose methods to bridge the gap between ontologies and the use cases they are intended to support.

## Methods

Annotations were taken from three diverse public domain datasets—(i) gene expression from the ArrayExpress Archive [5] and Gene Expression Atlas [6], (ii) the Europhenome mouse-specific databases [7-9], and (iii) the multi-species radiobiology database ERA-PRO [10,11]. These were selected to provide a set of representative data from functional genomics studies. The use cases provided data for matching anatomical terms to terms in two major anatomical ontologies, the Foundational Model of Anatomy (FMA) and a species-agnostic ontology, Uberon [12].

### Ontologies

The FMA is a symbolic representation of human anatomy, containing over 75,000 concepts over a range of granularity (http://sig.biostr.washington.edu/projects/index.html#FMA). Uberon is a multi-species metazoan anatomy ontology with the goals of: (1) supporting translational research by allowing comparison of phenotypes across species, and (2) providing logical cross-product definitions for GO biological process terms [13]. These ontologies were chosen for comparison on the basis of size (thus, likelihood of coverage) and importance in the anatomy ontology community.

### Data preprocessing

The ArrayExpress Gene Expression Archive currently contains over 615,000 assays, covering approximately 1200 different species. Of these assays, those which meet a particular standard of quality and clarity are re-annotated with ontology classes, summarized, and stored in the Gene Expression Atlas, which currently contains 138,000 assays from 30 species. Europhenome is an online resource developed to capture phenome data derived from mice using the standardized tests contained in EMPReSS (the European Mouse Phenotyping Resource of Standardised Screens) [7-9]. The ERA-PRO database is a legacy database of the results of radiation exposure of a wide range of organisms. It contains data on more than 300,000 animals from experiments conducted in Europe, the USA and Japan from 1954-1996 [10,11].

Ontology-curated annotations from the Atlas and non-standardized ones from the Archive, along with anatomy terms from Europhenome and ERA-PRO, constituted the sample of terms that we compared against the ontologies, as shown in Table 1.

**Table 1:** Anatomical and species data. Sample of anatomical and species data used for matching against the FMA and Uberon.

animal cap	Xenopus laevis
annulus fibrosus	Bos taurus
anterior cingulate cortex	Homo sapiens
anterior cingulate cortex	Pan troglodytes
anterior inferior parietal lobule	Homo sapiens
anterior inferior parietal lobule	Pan troglodytes

There were 1537 terms in our initial sample of anatomical structures by vertebrate species because once the structures were made species-agnostic, the terms from Europhenome and ERA-PRO were identical to terms in the Gene Expression Atlas, and thus did not increase that total number. These terms were predominantly human, mouse, and rat anatomical terms, but other species were represented as well, as presented in Table 2.

**Table 2:** Terms by species. Absolute number and percentage of terms by species (n = 1537).

Species	Number of anatomical terms	Percentage of total
*Homo sapiens*	782	50.88%
*Mus musculus*	429	27.91%
*Rattus norvegicus*	163	10.61%
*Bos taurus*	26	1.69%
*Pan troglodytes*	25	1.63%
*Gallus gallus*	23	1.50%
*Danio rerio*	18	1.17%
*Xenopus laevis*	16	1.04%
*Macaca mulatta*	13	0.85%
*Sus scrofa*	12	0.78%
Other vertebrate species	30	1.94%

Although these represent the set of terms as they were actually submitted by the users who annotated the use cases, we performed a small amount of manual and semi-automated preprocessing. The terms were normalized prior to matching as follows: species names were removed from the anatomical structures to be searched. Anatomical structure terms that had been unique only because they had different species names were resolved into a single term to remove duplicates. This practice may have increased the risk of false positives; however, we did not formally investigate that possibility. Inconsistent use of spaces within terms was resolved. Obvious misspellings and typographical errors, inconsistent punctuation and hyphenation were resolved. Anglicisms were converted to American spellings.

This preprocessing was carried out in order to reduce the number of false positive matches due only to duplication, and the number of false negative match failures due to: 1) Obvious errors in the data resulting in “terms” that would not exist in any anatomy ontology, 2) duplications due to different use of spaces, sentence casing, or punctuation and hyphenation, and 3) the emphasis of both the FMA and Uberon on American spellings as the primary term.

Whilst this is not strictly verbatim annotation data, this preprocessing represents simple data cleanup for which tools are readily available, so we consider it a representative model of how data would actually be submitted for matching against an ontology after automatic cleanup. After preprocessing, 1097 terms of the original 1537 remained. Inspection established that 229 terms exactly matched terms or synonyms in FMA, and 277 exactly matched terms or synonyms in Uberon. We used these matches as test controls before comparing the dataset to the ontologies.

### Comparison tools

Zooma is an automatic ontology mapping application developed at the European Bioinformatics Institute (EBI) (http://zooma.sourceforge.net/). Given a list of terms and an ontology in OWL/OBO format, or an ontology service to match against, Zooma can automatically map terms from the list to ontology terms. It can re-use existing mappings *e.g.*, from a database, discover mappings for new terms, and search ontologies to propose mappings against terms not mapped to a reference set. It produces two time-stamped tab-delimited text files, one containing successful matches, and one containing matching failures. Zooma delegates all ontology term fetching to the OntoCat library (http://www.ontocat.org). This library provides a uniform interface to query heterogeneous ontology resources including local ontologies in OWL or OBO as well as public ontology repositories, such as NCBO BioPortal and EBI Ontology Lookup Service.

Ontology Mapper [14] (http://www.ebi.ac.uk/efo/tools) is a Perl module, based on the Metaphone and Double Metaphone algorithms [15], that normalizes the terms in the list and the ontology to a representation of their sounds, and carries out the matching of similar-sounding terms. It performs exact matches without intervention, and proposes single or multiple approximate matches to the user for curator driven matching. Our dataset was run once against both the FMA and Uberon using Zooma, and once against both ontologies using Ontology Mapper.

These two approaches were used in order to identify particular issues that the user would confront in matching terms from annotations to terms in the ontologies. Because Zooma and Ontology Mapper use complementary approaches, we tested the ontologies with both tools in order to get more thorough coverage of potential issues.

## Results

A full set of results is available at http://www.ebi.ac.uk/~raven/uberon-fma-annotations-test/all-data.pdf .

**Figure 1:**
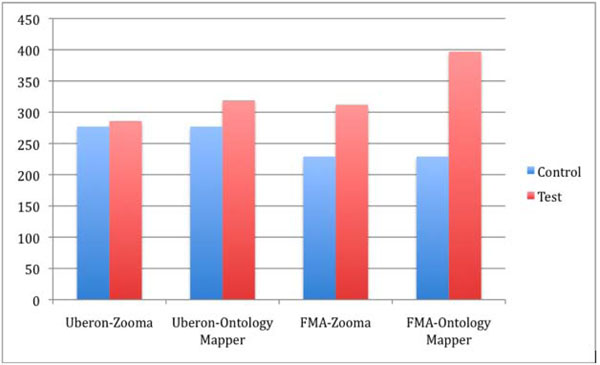
**Results by ontology and by tool.** Successful number of mappings (y-axis) in red, compared to control (blue) for Zooma on Uberon and FMA, and Ontology Mapper on Uberon and FMA.

As shown in Figure 1, when run with the term list against Uberon, Zooma provided 286 matches, compared to the control (visual inspection) of 277 exact matches. Ontology Mapper returned 319 matches, with user curation. For the FMA, Zooma returned 312 matches, and Ontology Mapper returned 397.

Precision/recall for each iteration are provided in Table 3.

**Table 3:** Precision and recall for matching results

	OM/FMA	OM/U	Z/FMA	Z/U
Precision	0.007	0.014	0.734	0.969
Recall	1.000	1.000	1.000	1.000

FMA=Foundational Model of Anatomy, OM=Ontology Mapper, U=Uberon, Z=Zooma

Confusion matrices are provided in Table 4.

**Table 4:** Confusion matrices for ontology term mapping

OM/FMA	True	False
Positive	229	32485
Negative	702	0

OM/U	True	False

Positive	277	19509
Negative	780	0

Z/FMA	True	False

Positive	229	83
Negative	787	0

Z/U	True	False

Positive	277	9
Negative	813	0

FMA=Foundational Model of Anatomy, OM=Ontology Mapper, U=Uberon,

This established that, for the terms covered in the ontologies, the tools returned a set of matchings from each ontology that was comparable to the curated set. although Ontology Mapper was particularly prone to false positives. However, the term coverage was quite low: 229 terms of 1099 anatomical terms used by annotators were found in the FMA (20.84%), and 277 terms were found in Uberon (25.20%). This precision and recall held only for the covered terms; both dropped to 0 for the large percentage of annotators' terms that were not represented. There was a 62.4% overlap between terms in the FMA and Uberon.

We examine the implications of these results in the Discussion.

## Discussion and conclusions

The first question that emerges from these results is why are there so few matches between such rich ontologies and real-life anatomical annotations from the user community? The tools we used to evaluate the ontologies provided complementary approaches to matching the data; as a result, a variety of issues were uncovered.

Some of the discrepancies are accounted for by the fact that the tools were designed for strict matching to reduce the number of false positives during automated lexical processing. However, the actual mismatches between the terms as the users provide them, and as they are represented in the ontologies accounted for much of the low hit rate. The strictness of the matching algorithm is a by-product of the fact that we wanted a low rate of false positives due to the known heterogenous nature of the input. The aim is to automate these processes of mapping in future and for the use cases we have, low false positives is important. In future work we can extend the use of methods to use more fuzzy matching techniques *e.g.*, Levenshtein distance-based methods, which will improve recall for adjectival forms.

### Annotation issues

The most obvious issues with the data that would block matching to the ontologies were standardized by preprocessing before the test was run. However, even after preprocessing, there were recurring problems that interfered with successful matching.

Most of the mismatches were due to annotations containing composite terms, where one or both of the terms, taken separately, actually would have matched. For example, *Liver/Kidney* does not match any entity in either ontology, but *Liver* alone and *Kidney* alone matched in both. Sometimes only one of the terms would have matched; for example, in *Acetabulum and pelvic soft tissues*, while *pelvic soft tissues* is too vague and would have required clarification, *Acetabulum* could have been an exact match. Tools that are able to split up the user’s annotations into individual anatomical terms, or even mine the user’s annotations for new anatomical terms, as well as the provision of searchable cross-products for compositional terms, can resolve some of these problems.

Sometimes it was unclear whether the composite term was actually referring to two different entities, or whether it was simply a redundancy, for example, *Adrenal cortex*, *adrenal gland*. Breaking down that composite term would have accomplished either one or two matches, depending on whether the user intended two different entities, or was using *Adrenal gland* redundantly to modify *Adrenal cortex*.

Occasionally the user would refer to a cell when it was obvious the sample referred to a tissue (example: *Adipocyte* vs. *Adipose tissue*), and whilst the cell name the user entered did not match, the intended tissue type actually would have.

Entities in anatomical ontologies are nouns for the most part, so when the user entered an adjective referring to the anatomical structure, such as *Abdominal* for *Abdomen*, or *Arterial* for *Artery*, the adjective would not match the ontology, yet was closely related to a term that was present.

Shorthand, such as *Antrum* for *Pyloric antrum*, or *Both ventricles* for *Left ventricle of heart* and *Right ventricle of heart* also kept the matching rate artificially low. Most of those examples could be expanded to full names of entities in the anatomy ontologies, but out of context, it was impossible to determine what a few meant, such as *Ventral* or *11 different tissues*. If a human curator cannot know what the annotation means, mapping becomes an impossible task for any automatic or curated tool.

Occasionally match failures occurred from the users’ utilization of named processes implying an anatomical location, such as *Colon pinch biopsy*, *BA* (*bronchioalveolar*) *lavage*, or *Bone marrow*, *flushed from femur*. The latter could be a composite term as well, although breaking it down into entities would require the tool to know how to deal with “flushed from”, in order to find the boundary between them.

### Ontology issues

Sometimes a mismatch would occur because the user made use of a synonym for a term that was actually in the ontology, but synonyms were missing. Sometimes an omission from an ontology was quite surprising—for example, the term *Anterior tibialis* was missing from Uberon and FMA. Addition of synonyms to both would improve utility.

The FMA and Uberon are designed for different purposes, so there is no consensus between them as to exactly what entities belong in an anatomical ontology. For example, *Alveolar macrophage* is included as a discrete entity in FMA, where Uberon does not contain it, and explicitly regards it as a composite to be generated from *Alveolus* and *Macrophage*, rather than belonging in the ontology itself. These differing definitions based on design decisions, while not apparent to the user, have an impact on whether that user’s terms can be expected to match terms in the ontologies being used.

Uberon handles embryological and non-human anatomical entities better than the FMA does. For this reason, Uberon performed relatively better in programmatically matching the heterogeneous data from this community.

### Mutual mismatch issues

The implicit assumption of the tools is that the mapping between list terms and ontology terms should be 1:1. This meant that there were sometimes approximate matches on a closely-related term, even in the absence of an exact match. For example, sometimes there was a superset or subset relationship approximate match, such as *Abdominal fat* and *Abdominal fat pad*, or *Fascia* and *Connective tissue*. Other proposed matches crossed levels of abstraction, for example, *Right lung* as opposed to *Lung*.

Additionally, a few cases of quantitative annotations, such as *75% kidney*, *25% liver* were present in the annotations; these will be ruled out of scope in future testing, as very few present ontologies can handle quantitative data. However, their presence does indicate a currently-unmet user need in data annotation.

Ontology classes tend to be expressed in the singular whilst annotations are written in singular and plural. Both tools did a better job of matching the singular terms than they did the same terms in plural; given the variation in working styles, tools that access ontologies for real-world applications will need to be better at dealing with singular-plural variation.

We have established that, although we were able to map almost half of the terms from the use cases to the ontologies, the process required a great deal of time, effort, and manual curation. There remains a vast gap between the way users use anatomical terms in free text annotation and the way they are represented in two of the richest anatomical ontologies. This exercise provided preliminary insight into the following issues:

• Which terms are available in which source(s)? Uberon was able to match more embryological and non-human terms in our data than the FMA did. This fact indicates the effect that the design decisions and scope of each ontology will have on users desiring to match their annotations with an ontology.

• Which areas require concentration in ontology development in order to obtain as much coverage as other areas have? Although they are the most difficult to represent rigorously in an ontology, and are consequently underrepresented in anatomical ontologies, brain terms and embryological terms make up the bulk of the annotation data. As a consequence, species-specific and cross-species anatomical ontologies need to represent that data, meaning that the particular difficulties of representing it need to be addressed.

• What maps, what does not map, and why? Simple terms such as *Liver*, *Kidney*, *Adrenal gland*, and *Retina* match very well in each ontology. Compositional terms, such as *Alveolar macrophage*, for example, tend to map poorly in each ontology, because of lexical similarity and granularity issues. Some of the compositional terms are to be expected in sampling (such as *Bone marrow from femur*); others are simply an artifact of combining samples or acquiring samples that cross multiple structures (*Liver*, *kidney*, *adrenal gland*, *adrenal cortex*); there needs to be a way of dealing with each scenario.

• What duplications and errors are our tools able to determine in the ontologies used in the comparison, and what suggestions would we make for additions and modifications to the source ontologies? We found omissions of surprisingly common terms and synonyms utilized in the user community, such as *Anterior tibialis* for "tibialis anterior". We also found that Uberon had the term *Ureter* twice, with two separate IDs, and requested a consolidation.

• What suggestions do we want to make to the tool developers for functionality that would make it easier for users to obtain better matches? Currently there is a large risk of false positives for matching. Lemmatisation and stemming would prevent some of the need for pre-processing, but would exacerbate the tendency to false positives. We would suggest tools refined more precisely to the specific needs of the anatomy domain. Misspellings, hybrid terms with elements of multiple languages, and other flawed input data present a challenge to the canonical terms in ontologies. The input data needs to be cleaned up and made consistent, a task that was done manually for this study, but which is prohibitive for scaling.

• The requirements for cross-products in many annotation use cases. Terms such as *Bone marrow from femur* failed to match, even though both *Bone marrow* and *Femur* were in the ontologies.

These insights will also inform our future efforts in developing and refining ontology-matching tools. Although the purpose of this study was not to evaluate the tools *per se*, some interesting findings emerged that will provide a basis for future refinement of the tools. Ontology Mapper was far more sensitive to potential mappings than the anatomist was, tending toward false positives such as proposing to match *Perisoteum of ilium* with *Whole embryo*. This was an artifact of its Double Metaphone algorithm. Zooma did not provide any false positives, as a result of its exact matching, but made many false negative errors. The discrepancy between Zooma's exact matches and the anatomist's is an interesting finding. The numbers should have been the same. Possible reasons for the discrepancy include human error, a possible bug in the code, or the presence of non-printing characters in the data, recognisable by Zooma but invisible to the anatomist. We will follow up on this in future work. Another issue for future work is aligning matches along semantic content rather than the lexical matching that sufficed for this exploratory study. The lexical matching approach worked for such general vertebrate structures as *Liver* or *Kidney*, but for annotations containing more detailed accounts of species-specific structures (*e.g.*, zebrafish *Frontal bone*, rat *Anterior prostate*), this approach will need to be refined in order to avoid another source of false positives.

In order for ontologies to realize their potential, they need to be used. The user must perceive the benefit from their use, whether that benefit takes the form of ease of data entry, time saved, and replacement of manual inspection with automation. The current state of anatomical ontologies leaves a gap between the needs of the user and which ontologies are available. There is a real and growing need for tools such as Zooma, Ontology Mapper, and others that can complement the functions of ontologies in bridging that gap, removing barriers between the ontology and the community of users it is intended to serve.

## Competing interests

The authors declare that they have no competing interests.

## Authors' contributions

RST participated in the design of the study, performed the data preprocessing, mappings, and analysis, and drafted the manuscript. TA developed Ontology Mapper and OntoCat. TB developed Zooma. MG provided annotation data for use in the mappings. JH provided annotation data for use in the mappings, and helped to draft the manuscript. AMM provided annotation data for use in the mappings. JM participated in the design of the study, and helped to draft the manuscript. PS provided annotation data for use in the mappings, and helped to draft the manuscript. HP conceived of the study, participated in its design, and helped to draft the manuscript. All authors read and approved the final manuscript.
